# Distal Radial Access for Perimembranous Ventricular Septal Defect Closure

**DOI:** 10.1016/j.jaccas.2025.106563

**Published:** 2026-01-21

**Authors:** Charlotte Johanna Cool, Achmad Fauzi Yahya, Dian Larasati Munawar, Melawati Hasan, Aninka Saboe, Achmad Fitrah Khalid, Rina Dhyanti Permatasari, Muhammad Munawar

**Affiliations:** a Department of Cardiology and Vascular Medicine, Faculty of Medicine, University of Padjadjaran, Bandung, Indonesia; b Binawaluya Cardiac Center, Jakarta, Indonesia

**Keywords:** distal radial access, percutaneous closure, ventricular septal defect, ventricular septal defect occluder

## Abstract

**Background:**

Percutaneous perimembranous ventricular septal defect (pmVSD) closure traditionally uses femoral arterial access, which carries risks of vascular complications and prolonged bed rest.

**Objective:**

We aim to report the first case series of pmVSD closure using distal radial access (DRA) with a single arterial retrograde approach.

**Methods:**

Eight consecutive adult patients underwent percutaneous pmVSD closure via DRA using the Amplatzer Ductal Occluder II between 2018 and 2024 across 2 centers. All procedures used a single arterial access with a retrograde approach.

**Results:**

Technical success was achieved in all patients (100%). The mean procedure time was 49.4 ± 7.3 minutes. Defect sizes ranged from 3 to 6 mm (right ventricle) and 5 to 6 mm (left ventricle). Complete closure occurred in all patients with no residual shunts at 1-year follow-up. No major complications occurred.

**Conclusions:**

DRA represents a safe, effective alternative for percutaneous pmVSD closure, offering reduced vascular complications, shorter hospital stays, and improved patient comfort compared with traditional femoral access.

## Graphical Abstract

**Figure:**
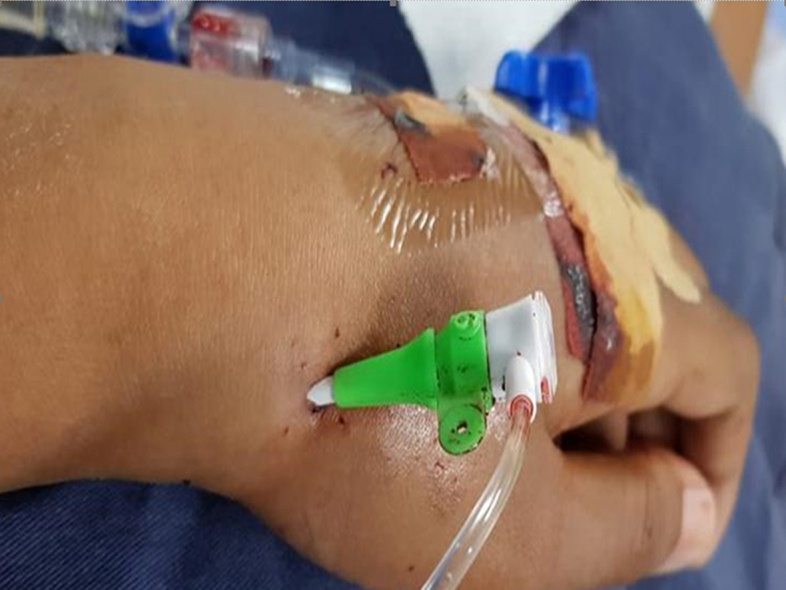

Ventricular septal defect (VSD) is the most common congenital cardiac anomaly, with the perimembranous VSD (pmVSD) accounting for approximately 80% of all VSDs. Transcatheter device closure has emerged as a widely accepted alternative to surgical repair, with the Amplatzer Ductal Occluder II (ADO II) demonstrating excellent results.^1^ Traditional approaches use femoral arterial access, which, although effective, carries risks of vascular complications, prolonged bed rest, and patient discomfort.^2^ Proximal radial access offers advantages but may result in radial artery occlusion in 5.5% to 6.4% of cases.^3^ Distal radial access (DRA) through the anatomical snuffbox offers a promising alternative, with fewer vascular events and greater patient comfort.

To our knowledge, this is the first published case series describing DRA for pmVSD closure with ADO II.

## Materials and Methods

This retrospective study included 8 consecutive patients who underwent transcatheter retrograde pmVSD closure across 2 centers between January 2018 and December 2024 (Table 1). All were young adults with an isolated pmVSD.

**Table 1:** Patient Characteristics, Procedures, and Follow-Up

Case No.	Sex	Age (y)	Right/Left Distal Radial	Radial Sheath Length	Defect Size From RV (mm)	Defect Size From LV (mm)	SAR (mm)	Aneurysm	Device Size (mm)	ACT Achieved >300 s	Fluoro Time	Contast Volume	Procedure Time (min)	During Procedure and 1-Year Follow-Up
1.	F	19	Left	Prelude, 6-F, 7 cm	5	6	9	Yes	6/6	Yes	24	70	63	NoC
2.	F	22	Right	Prelude, 6-F, 7 cm	3	6	8	No	6/4	Yes	22	64	45	NoC
3.	M	27	Left	Prelude, 6-F, 7 cm	5	6	7	Yes	6/4	Yes	22	67	52	NoC
4.	F	34	Right	Prelude, 6-F, 7 cm	5	6	6	No	6/4	Yes	24	64	45	NoC
5.	F	22	Left	Prelude, 6-F, 7 cm	3	5	7	No	6/6	Yes	25	70	55	NoC
6.	F	36	Right	Prelude, 6-F, 7 cm	4	5	8	No	6/4	Yes	24	70	40	NoC
7.	M	29	Right	Glidesheath 6-F, 10 cm	5	6	6	No	6/4	Yes	31	75	45	NoC
8.	M	27	Right	Glidesheath 6-F, 10 cm	5	6	7	No	6/6	Yes	33	75	50	NoC

LV = left ventricle; NoC = no complications; RV = right ventricle; SAR = subaortic rim.

Inclusion criteria were defect <6 mm, subaortic rim >3 mm, left-to-right shunt confirmed by transesophageal echocardiography (TEE), and the absence of aortic valve prolapse or regurgitation. Patients with other structural heart diseases requiring surgery were excluded. All patients had palpable distal radial pulses and provided written informed consent. The protocol was approved by the institutional review board (IRB No. LB.02.01/X.6.5/378).

## Procedure and Results

The procedure was performed under general anesthesia and TEE guidance. The patient's hand was placed in pronation across the abdomen to expose the anatomical snuffbox. After local anesthesia with 3 to 5 mL of xylocaine, the distal radial artery was punctured with a 21-gauge needle at a 30° to 45° angle from lateral to medial. The choice of right or left DRA was operator-dependent.

**Figure 1:**
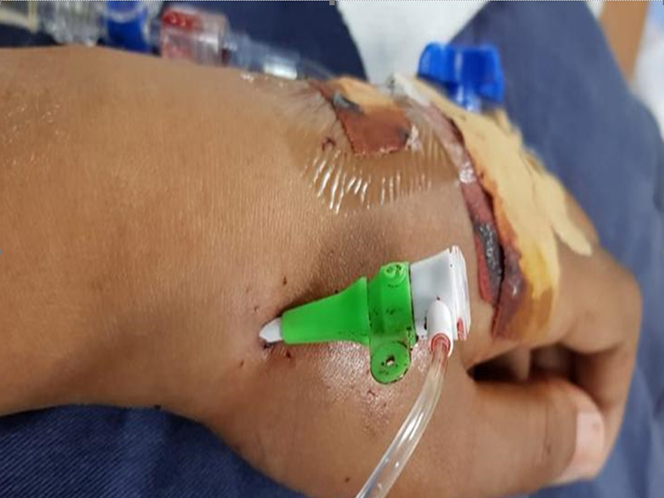
Distal Radial Access Distal radial access using a 6-F hydrophilic-coated radial sheath. The forearm prone position is more comfortable for the patient.

After successful puncture, a 0.018-inch guidewire and a 6-F hydrophilic sheath were introduced (Figure 1). A mixture of 5,000 U heparin, 100 μg of nitrate, and 10 mL of saline was administered to prevent spasm. Activated clotting time was maintained at >300 seconds.

**Figure 2:**
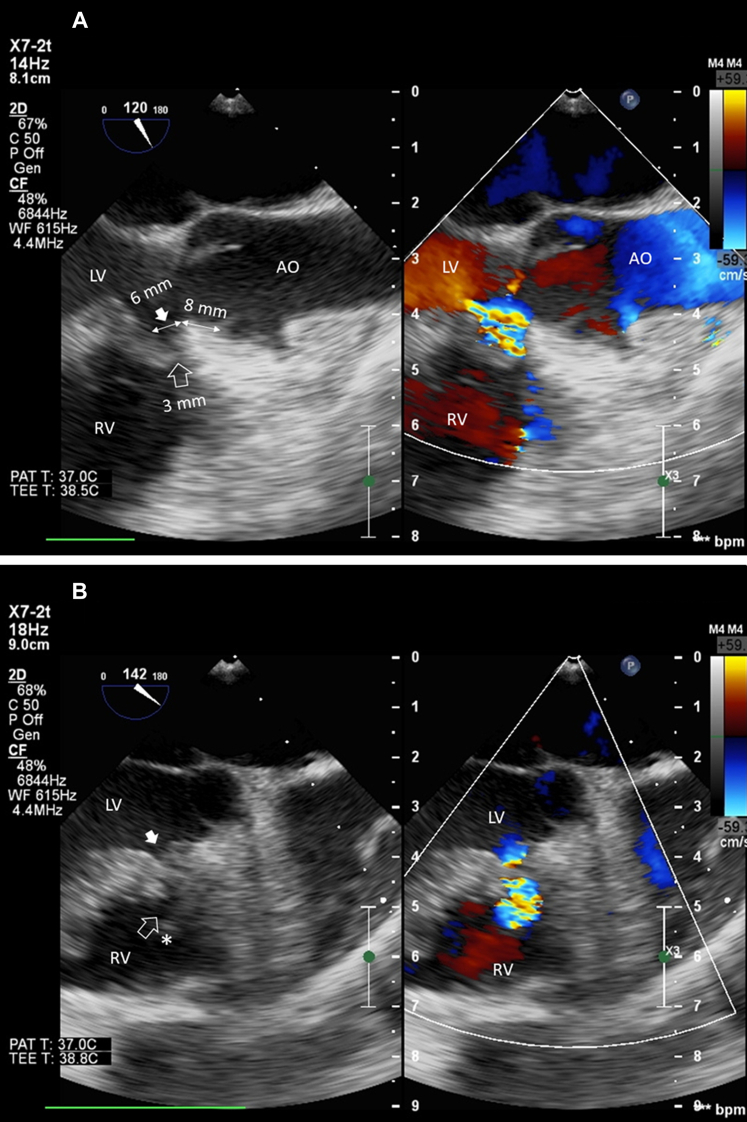
Transesophageal Echocardiography Assessment of the Perimembranous Ventricular Septal Defect (A) Patient 2: Measurement of the defect size. From the left ventricular (LV) site (inlet, solid arrow), the defect size was 6 mm, and from the right ventricular (RV) site (outlet, hollow arrow), it was 3 mm. The subaortic rim was 8 mm. No aneurysm was observed. In the right panel, the turbulence flow was clearly seen through the defect during systole. (B) Patient 1: A clear image of an aneurysm (asterisk) and defect inlet (solid arrow) and outlet (hollow arrow). AO = aorta.

**Figure 3:**
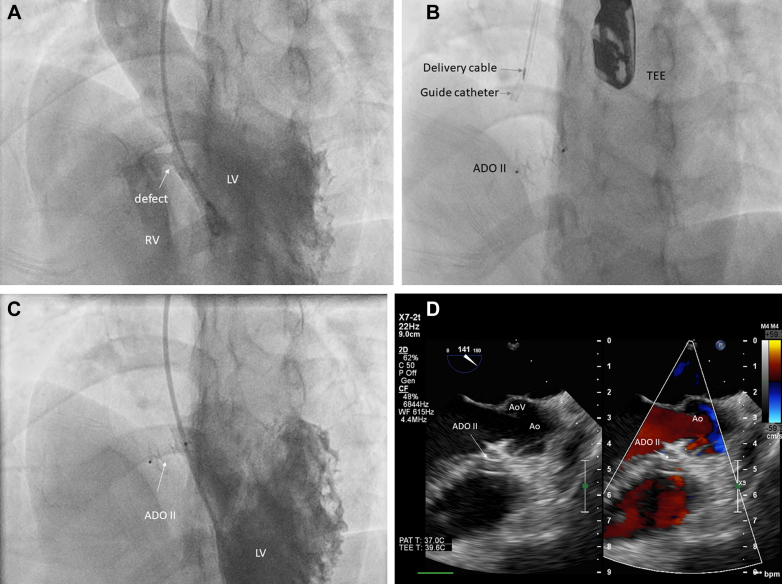
Fluoroscopy During Perimembranous Ventricular Septal Defect Closure Via Distal Radial Access Left ventriculography using a pigtail catheter at 45° left anterior oblique and 25° cranial views. (A) A left-to-right shunt was observed, and the size of the defect was 6 mm. (B) The ADO II device was released from the delivery cable. (C) Postprocedure ventriculography, with no leakage observed. (D) Transesophageal echocardiography (TEE) confirmed that there was no leakage at the defect. ADO II = Amplatzer Ductal Occluder II; Ao = aorta; AoV = aortic valve; LV = left ventricle; RV = right ventricle.

Hemodynamic data from the right heart were obtained from transthoracic echocardiography (TTE). The diameter of the VSD and surrounding tissue (such as the subaortic rim) was measured on the left ventricular side using TEE (Figures 2A and 2B). The ADO II device, approximately 1 mm larger than the smallest VSD diameter, was chosen. Left ventriculography was performed using a pigtail catheter at the left anterior oblique at 45° and cranial at 25° views (Figure 3A). The 6-F Judkins Right Vista Brite (0.070-inch internal diameter, Cordis) guiding catheter and a 0.035-inch, 180-cm angulated hydrophilic Glidewire (Terumo Corporation) were inserted through DRA. The VSD was easily crossed from the left ventricle using the 6-F Judkins Right guide catheter with a mounted glidewire under fluoroscopy guidance. The glidewire was parked in the pulmonary artery or right ventricle (RV). The guide catheter was then advanced over the glidewire into the RV, and the glidewire was withdrawn from the guide catheter. Under fluoroscopy and TEE guidance, the ADO II was then inserted into the guide catheter (as a delivery catheter) and pushed further into the distal guide catheter. The distal disk was deployed at the RV and brought into the right side of the pmVSD. The proximal part of the disk was then deployed at the left side of the pmVSD. TEE was performed to check for any encroachment on the aortic valve, aortic regurgitation, and residual left-to-right shunting. No conduction disturbances were noted in any of the cases. A vigorous “tug test” was performed to test the stability before releasing the device. After the device was deployed (Figure 3B), TEE was performed to verify the device's alignment within the ventricular septum and assess any potential disruption to the aortic valve (Figure 3C). At the end of the procedure, left ventricle angiography was performed to check for residual shunt across the VSD (Figure 3D). The sheath was removed, and a hemostatic band was applied.

The characteristics of patients, including their defects, the size of the device, and the procedure time, are presented in Table 1. Among the 8 patients, 3 were male, with ages ranging from 19 to 36 years. The diameter of the defect was 3 to 5 mm from the RV and around 6 mm from the left ventricle. The thickness of the defect was 4 to 5 mm. The subaortic rim was 7 to 9 mm. There were 2 cases with an aneurysm. The ADO sizes were 6/4 to 6/6 mm. No single complication was noted. After the procedure, all patients were monitored in the hospital and discharged the following day. All patients were administered acetylsalicylic acid, 100 mg once daily, for 6 months. Follow-up visits were arranged for the subsequent 1, 3, 6, and 12 months.

**VISUAL SUMMARY:** Timeline and Workflow of the Case

Date/Stage	Events and Key Findings
Initial evaluation	Adult patients presented with an isolated perimembranous ventricular septal defect (pmVSD). Transthoracic echocardiography (TTE) and transesophageal echocardiography (TEE) confirmed a pmVSD with a left-to-right shunt, adequate subaortic rim (>3 mm), and no aortic regurgitation.
Procedure day	Palpable distal radial pulse. Left or right distal radial access was selected at the operator's discretion. Under general anesthesia, distal radial artery (DRA) access was obtained via the anatomical snuffbox using a 6-F hydrophilic sheath. A single retrograde approach was performed using a Judkins Right guiding catheter and hydrophilic glidewire to cross the defect. The Amplatzer Ductal Occluder II device was deployed retrogradely under TEE and fluoroscopic guidance. Activated clotting time maintained >300 seconds. No procedural complications or conduction disturbances were observed. Hemostasis achieved with a compression band at the DRA site. No crossover to femoral access.
Postprocedure (days 1-2)	Patient was monitored overnight with continuous electrocardiogram (ECG) telemetry; no rhythm disturbances or access-site complications occurred. Discharged the next day on acetylsalicylic acid 100 mg daily for 6 months.
Follow-up month 1	Clinical examination normal; ECG and TTE showed stable device position, no residual shunt, no arrhythmia, and patent distal radial pulse on palpation.
Follow-up months 3-6	All patients remained asymptomatic; echocardiography confirmed complete closure and normal ventricular function. No conduction abnormalities or device-related complications noted.
Follow-up month 12	At 1-year follow-up, all 8 patients demonstrated sustained complete closure, preserved conduction, and no vascular or thromboembolic events.

Each follow-up visit included a detailed physical examination, an electrocardiogram, and TTE. During follow-up visits, all patients were in good condition, with no murmur detected, no cardiac conduction disturbances, and no total atrioventricular block observed. TTE was performed during the follow-up visits and revealed that all devices were well seated, with no leakage detected.

## Discussion

This case series represents the first published experience with DRA for percutaneous pmVSD closure, demonstrating excellent technical success and safety outcomes. The prior published case report of pmVSD closure used proximal radial access.^4^ The DRA approach offers several advantages over traditional femoral access, including reduced vascular complications, immediate patient mobilization, and improved comfort.^5^ Although the distal radial artery is approximately 20% smaller than the proximal radial artery, with a mean diameter of 2.31 to 2.35 mm, DRA has demonstrated high success rates in large contemporary cohorts—around 93% to 95%—with or without ultrasound screening or guidance.^5^^,^^6^ As compared with conventional radial access (proximal radial access), DRA is associated with shorter hemostasis times and lower rates of radial artery occlusion (risk ratio: 0.38), bleeding, and pseudoaneurysm, though at the expense of slightly longer access times and higher crossover rates.^5^

Key technical considerations include proper patient positioning, adequate anticoagulation, and careful device selection based on defect morphology. The single arterial access retrograde approach simplifies the procedure while maintaining safety and efficacy.^7^

Patient selection criteria include an adequate subaortic rim (≥5 mm), an appropriate defect size for the ADO II device, and suitable radial artery anatomy. Contraindications include severe peripheral arterial disease, inadequate subaortic rim, or extremely large defects requiring larger delivery systems.

The learning curve for DRA in structural interventions requires experience with radial access techniques and familiarity with ADO II device characteristics. Operator comfort with both radial access and pmVSD closure is essential for optimal outcomes.

Complete atrioventricular block after transcatheter perimembranous VSD closure occurs in approximately 0.5% of cases in recent large series.^8^ Despite the low rate, the risk is clinically important because of the possible need for pacing or late occurrence. In our 8-patient DRA series, no conduction disturbances were observed during 1-year follow-up.

Invasive Q_p_/Q_s_ and pulmonary vascular resistance measurements were not performed, because all patients had small restrictive pmVSDs with clear left-to-right shunts and normal right-sided pressures on echocardiography. Device closure was undertaken in small restrictive perimembranous VSDs that demonstrated either left ventricular volume overload or the presence of aortic regurgitation.^9^^,^^10^

Limitations include that this study represents only 2 centers, is a retrospective, nonrandomized case series with a small sample size, and lacks a control group, which limits the generalizability of the findings. Although all eligible patients were included during the study period, the potential for selection bias remains. Objective assessment of radial artery patency (such as Doppler ultrasound or Barbeau testing) was not performed systematically before or after the procedure, so subclinical vascular complications may have been missed.

Conduction monitoring was limited to physical examination and serial 12-lead electrocardiogram during follow-up visits; there was no use of continuous inpatient telemetry or ambulatory Holter monitoring after discharge. This could underestimate the incidence of transient or late-onset conduction disturbances, including atrioventricular block. Follow-up was limited to 1 year, and a longer surveillance period is warranted to assess for delayed complications or recurrences.

Finally, the technique requires considerable operator experience and may not be generalizable to all centers or patients, especially where DRA expertise or equipment is limited.

## Funding Support and Author Disclosures

The authors have reported that they have no relationships relevant to the contents of this paper to disclose.

**Take-Home Messages**

• DRA provides a safe and effective alternative to femoral access or proximal radial access for percutaneous pmVSD closure with reduced vascular complications and improved patient comfort.
• A single arterial access retrograde approach via DRA maintains high technical success rates while offering immediate patient mobilization and shorter hospital stays.
• Careful patient selection, adequate operator experience with radial techniques, and proper device selection are essential for optimal outcomes in DRA-based pmVSD closure.
